# Polar Ruddlesden–Popper Rare-Earth Aluminum Oxynitrides with Partial Anion Order

**DOI:** 10.1021/acs.inorgchem.6c03989

**Published:** 2026-10-13

**Authors:** Judith Oró-Solé, Jhonatan R. Guarín, Ignasi Fina, Carlos Frontera, Jaume Gàzquez, Saul Estandia, Mehrdad Ghiasabadi Farahani, Clemens Ritter, Pablo Loza-Alvarez, Gustavo Castro-Olvera, Josep Fontcuberta, Amparo Fuertes

**Affiliations:** † 54449 Institut de Ciència de Materials de Barcelona (ICMAB-CSIC), Carrer dels Til.lers, 08193 Cerdanyola del Vallès, Spain; ‡ Institut Laue-Langevin, 71 Av. de Martyrs, BP 156, F-38042 Grenoble Cedex 9, France; § ICFO-Institut de Ciències Fotòniques, The Barcelona Institute of Science and Technology, 08860 Castelldefels, Spain

## Abstract

Anion order in heteroanionic compounds may induce electrical dipoles and the formation of noncentrosymmetric polar structures. The majority of perovskite oxynitrides are centrosymmetric, but some of them show high dielectric permittivities and ferroelectric response resulting from local anion order. Here, we report a study of polar, partially anion-ordered Nd_2_AlO_3_N and Sm_2_AlO_3_N *n* = 1 Ruddlesden–Popper oxynitrides, prepared by solid-state reaction in flowing N_2_ at 1500 °C. They crystallize in the noncentrosymmetric *I*4*mm* space group with parameters *a* = 3.703314(3) and *c* = 12.545382(14) Å for Nd_2_AlO_3_N and *a* = 3.674477(5) and *c* = 12.40414(3) Å for Sm_2_AlO_3_N. Neutron powder diffraction data of Nd_2_AlO_3_N indicate that oxygen occupies the equatorial sites 4b of the AlO_5_N octahedra, whereas both anions occupy two crystallographically independent 2a axial X2 and X3 sites, with different O/N populations (68/32 for X2 and 32/68 for X3) and a larger bond distance to the Al atom for the oxygen rich site. Dielectric permittivities of 260 and 150 are measured for Nd_2_AlO_3_N and Sm_2_AlO_3_N, respectively, and second-harmonic generation spectroscopy and scanning transmission electron microscopy furnish complementary evidence of the polar nature of both compounds. These results represent the first experimental demonstration of acentric functionalities in a Ruddlesden–Popper oxynitride.

## Graphical abstract

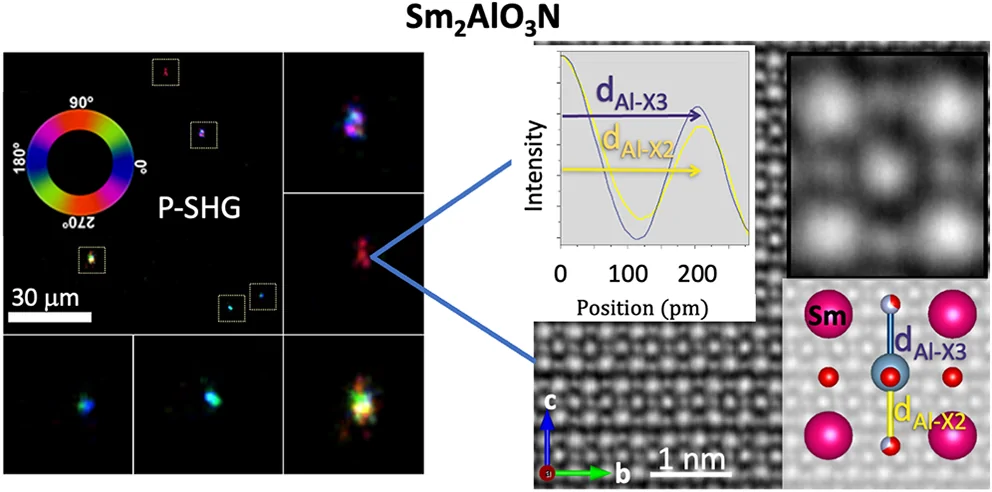

## Introduction

Perovskite oxynitrides have been widely investigated because of their magnetic, photocatalytic, and dielectric properties.^1–4^ An important feature in oxynitrides and in other heteroanionic compounds, such as oxyhalides and oxychalcogenides, is the arrangement of the two anions that may affect the physical properties, as it determines differences in bond covalency and bond distances around the cations.^5–8^ Anion order may generate bond asymmetries and has special relevance for the existence of permanent dipoles and acentric structures, leading to high dielectric permittivities and ferroelectric response.^7^ If the two anions have different charges as in the case of oxynitrides, they are prone to order if distinct crystallographic sites exist according to Pauling’s second crystal rule (PSCR),^6,9^ which states that in a given position, the charge of each anion (*q*) tends to compensate the strength of the electrostatic valence bonds (*b*) reaching it from the cations to which it is coordinated, calculated as

1 $$b=\sum_{i}\frac{z_{i}}{\nu_{i}}$$

where *z_i_* is the electric charge of each cation bonded to a given anionic position and ν*_i_* is the anion coordination number.

In the group of simple oxynitride perovskites with the formula ABO_3–*x*_N_*x*_ (A = alkaline earth metal, rare earth metal; B = transition metal), all the anion sites show similar coordination environments with the cations (hence the same *b*), which lead to O/N disorder for the majority of compounds, and for some cases, as in LaTaON_2_, different symmetries and anion distributions have been reported depending on the method of preparation.^10^ In pseudocubic perovskites, it is generally accepted that the local order of nitrogen in a *cis* configuration in the B octahedra is stabilized because of the larger covalency of metal–nitride bonds compared to metal–oxide bonds.^11–13^ Such local order has been suggested to be the origin of ferroelectric order in SrTaO_2_N^14^ and BaTaO_2_N,^15^ which have been described in centrosymmetric space group average structures. In the polar hexagonal perovskite of 6H polytype BaWON_2_, nitrogen and oxygen order in corner- and face-sharing positions, respectively, of two WO_3_N_3_ face-sharing octahedra, whereas nitrogen occupies the anion positions of the corner-sharing WN_6_ octahedra.^16^ Anion order, together with second-order Jahn–Teller effects and electrostatic repulsions between highly charged W^6+^ ions, stabilizes a noncentrosymmetric structure and induces a spontaneous polarization.

Ruddlesden–Popper oxynitrides (AX)­(ABX_3_)_n_ (X = O, N) formed by stacking of n perovskite blocks and rock salt-type layers^17^ have been obtained for a limited number of stoichiometries. Reported n = 1 members are R_2_AlO_3_N (R = La, Nd, Sm),^18^ Sr_2_TaO_3_N, Ba_2_TaO_3_N,^19^ and very recently R_2_TaO_3+*x*_N_2–*x*_ (R = La, Nd, Sm, Eu).^20^ The *n* = 1 and *n* = 2 members of the series SrO­(SrNb­(O,N)_3_)_n_ ^21^ were also obtained, and tantalum compounds with high n have been reported with compositions Eu_3_Ta_2_O_3_N_4_,^22^ Eu_3_Ta_2_ON_5_,^23^ A_2_LaTa_2_O_6_N_2_ (A = Li, Na, K; Rb) (*n* = 2),^24^ and A_2_Ca_2_Ta_3_O_9_N (*n* = 3).^25^ In contrast with simple oxynitride perovskites, in all of these layered oxynitride structures, there are crystallographically independent anion sites that show different topologies in their coordination polyhedra, favoring the ordered distribution of oxygen and nitrogen. In Ruddlesden–Popper *n* = 1 compounds, there are two environments for anions at equatorial and axial sites of the BX_6_ octahedra. For a divalent cation such as Sr or Ba at the A site and Nb^5+^ or Ta^5+^ at the B site, the bond strength sums calculated with eq [1] are *b* = 2.55 for equatorial anion positions of the BX_6_ octahedra and 1.94 for the apical sites. In the case that A is a trivalent rare-earth cation and B = Ta^5+^, the corresponding *b* values are 3.0 for the equatorial sites and 2.5 for the apical positions. Neutron diffraction studies of Sr_2_TaO_3_N,^26^ Ba_2_TaO_3_N,^27^ and Sr_2_NbO_3_N^28^ indicate that oxygen occupies the axial sites, whereas the equatorial sites are occupied by 50% of each anion, which completely agrees with the expected distribution using PSCR.^6^ In contrast, for Ce_2_TaON_3_, PSCR predicts the total occupancy of N in equatorial sites and 50% O/N on the axial sites, as it is experimentally confirmed by neutron diffraction.^20^

The rare-earth aluminum oxynitrides R_2_AlO_3_N (R = La, Nd, Sm) were first prepared by Roger Marchand in a sealed tube from mixtures of rare earth oxide and AlN at 1350 °C. Their crystal structures were not determined, but they were described in the *I*4/*mmm* aristotype of *n* = 1 Ruddlesden–Popper compounds, with cell parameters in the ranges of *a ≈* 3.7–3.8 Å and *c* ≈ 12.4–12.8 Å (Figure 1a) depending on the rare earth.^18^ More recently, Nd_2_AlO_3_N and Sm_2_AlO_3_N were prepared by the reaction between Nd_2_O_3_ or Sm_2_O_3_ and Al_2_O_3_ and C under flowing N_2_ at 1250 °C.^29^ A Rietveld refinement from time-of-flight neutron powder diffraction data of Nd_2_AlO_3_N prepared by the first procedure was performed in the noncentrosymmetric structure *I4mm*, indicating total anion order with 100% of oxygen in the equatorial sites (4b) and one of the axial sites and nitrogen occupying the second axial site (2b) (Figure 1b).^30^ The two axial sites in this compound (as well as in in the phases of La and Sm) are topologically equivalent and show the same PSCR bond strength sum, 2.17;^6^ hence, total order of anions is not expected. The bond strength sum for the equatorial sites is close to this value, 2.33. The reported *I4mm* structure of Nd_2_AlO_3_N,^30^ with two different axial Al–N and Al–O bond distances, belongs to the C category of noncentrosymmetric materials described by Halasyamani and Poeppelmeier^31^ that show pyroelectricity, piezoelectricity, and SHG response, representing the first example of a polar Ruddlesden–Popper oxynitride, although these symmetry-dependent properties have not been investigated.

**Figure 1:**
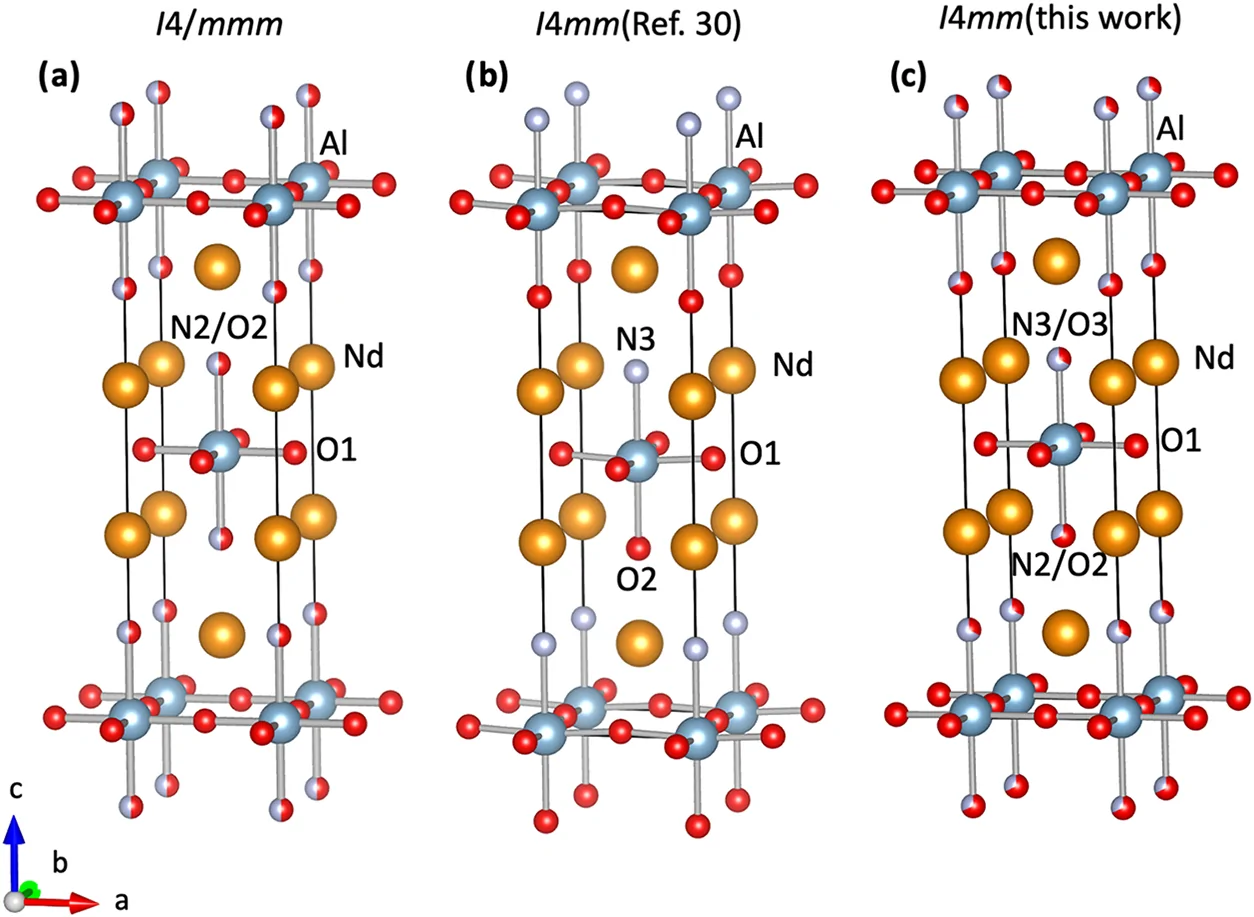
Structure models of Nd_2_AlO_3_N. (a) Centrosymmetric *I*4/*mmm* model. (b, c) *I*4*mm* crystal structures reported by R.Marchand^30^ and in this work, respectively.

Here, we report an alternative synthesis of Nd_2_AlO_3_N and Sm_2_AlO_3_N in flowing N_2_ at 1500 °C, the determination of the crystal structures of the two compounds from synchrotron X-ray powder diffraction data and from neutron powder diffraction data for the Nd compound, and the study of their dielectric properties and second-harmonic generation behavior. Rietveld refinement of high-resolution neutron powder diffraction data of Nd_2_AlO_3_N performed in the *I*4*mm* space group indicates that the equatorial sites of the Al octahedra are occupied by oxygen, whereas, as predicted by PSCR, there is some disorder of nitrogen and oxygen in the two axial sites, in contrast with the previous study.^30^ This distribution leads to differences in the apical bond distances of c.a. 0.15 Å in both Nd_2_AlO_3_N and Sm_2_AlO_3_N that are directly observed in iDPC-STEM (integrated differential phase contrast-scanning transmission electron microscopy) images of microcrystals. The preferred occupancy of the anions in the axial sites induces the noncentrosymmetric structure, and in consequence, a second-harmonic generation (SHG) response is observed. The dielectric permittivity is as high as 150 for Sm_2_AlO_3_N and 260 for Nd_2_AlO_3_N. These findings represent the first study of symmetry-dependent properties of the only two examples of polar Ruddlesden–Popper oxynitrides. In connection with the investigation of anion order and local structures, our results expand the possibilities of design of new heteroanionic polar materials.

## Experimental Methods

### Synthesis and Chemical Characterization

A sample of 500 mg of Nd_2_AlO_3_N was prepared by solid-state reaction between Nd_2_O_3_ (Sigma-Aldrich, 99.99%), NdN (Alfa Aesar, 99.9%), and AlN (Sigma-Aldrich, 99.8%) in molar ratios of 0.85:0.3:1.0 under flowing N_2_ (Air Liquide, 99.9999%) using four treatments of 12 h at 1500 °C with intermediate regrinding and pelletizing. The synthesis of 500 mg of Sm_2_AlO_3_N was performed starting with Sm_2_O_3_ (Sigma-Aldrich, 99.998%) and AlN (Sigma-Aldrich, 99.8%) using the same gas and one single cycle of 12 h at 1500 °C. The rare earth oxides were previously treated at 900 °C under a vacuum of 10^–3^ Torr during 12 h for dehydrating. Handling of the reactants, mixing, and pelletizing were performed in a glovebox under recirculating Ar. The pellets were placed in a molybdenum crucible in a high-temperature tubular furnace using a zirconium foil close to the sample to scavenge H_2_O and O_2_ impurities from the purging gas (below 0.5 and 0.1 ppm, respectively). The reaction tube was evacuated to 10^–3^ Torr and purged three times with N_2_, then heated at 300 °C/hour up to 1500 °C, kept at this temperature for 12 h, and cooled to room temperature. The obtained samples were stable in air. For electrical measurements, pellets of 5 mm diameter were prepared from the obtained powders, by treatment at the same temperature during 3 h. Nitrogen analyses were performed in a Thermo Fisher Scientific instrument heating the samples up to 1060 °C under O_2_, using MgO, WO_3_, and Sn as additives and atropine as a reference standard. Cation compositions were analyzed by EDX in an FEI Quanta 200 FEG microscope equipped with an EDAX detector with an energy resolution of 132 eV.

### Structural Characterization

Laboratory X-ray powder diffraction patterns were acquired on capillaries of 0.3 mm diameter using a Bruker D8 Advance A25 diffractometer in a Debye–Scherrer configuration with Mo Kα_1_ radiation (λ = 0.7093 Å). High-angular resolution synchrotron X-ray powder diffraction data were collected on capillary samples at the MSPD beamline of the Alba synchrotron (Cerdanyola del Vallès, Spain),^32^ using 30 keV energy with λ = 0.41417 Å and 2.0° ≤ 2θ ≤ 59° for Nd_2_AlO_3_N and 0.41376 Å and 3.0° ≤ 2θ ≤ 50° for Sm_2_AlO_3_N. Neutron powder diffraction data for Nd_2_AlO_3_N were collected on the D20 high-intensity diffractometer at the Institut Laue–Langevin (ILL), France, using a vanadium can as a sample holder. The pattern was measured with a short wavelength of 1.37 Å by using 118° take-off angle, yielding high resolution. Rietveld analysis was performed using the program Fullprof.^33^

Electron diffraction patterns were obtained in a JEOL 1210 transmission electron microscope operating at 120 kV using a side entry sample holder with a double tilt, ±60°/±30°. The samples were prepared by depositing the powder on carbon-coated copper grids.

Atomic-scale imaging study was carried out at the Joint Electron Microscopy Center at ALBA (Cerdanyola del Vallès, Spain) with an aberration-corrected Thermo Fischer Scientific Spectra 300 electron microscope operated at 200 kV and equipped with a Schottky field emission gun (X-FEG). Integrated differential phase contrast (iDPC) STEM imaging was used to obtain atomic-resolution images of Nd_2_AlO_3_N and Sm_2_AlO_3_N nanocrystals. The STEM-iDPC images were analyzed using a custom Python script to identify atomic column positions and calculate interatomic distances (see Figure S1 for details of the calculation).

### Optical and Electrical Measurements

Diffuse reflectance spectra were registered at room temperature on a JASCO V-780 UV–visible/NIR spectrophotometer, with an operational range of λ = 200–1600 nm. Capacitance was measured on sample pellets using an AC excitation signal of 1 V at various frequencies (*f* = 1 kHz – 1 MHz), using an LF4182 impedance analyzer (Agilent Co.). Dielectric permittivity was determined on sintered pellets from the capacitance data using nominal capacitor geometry (area (*A*) and thickness (*t*)) and *C* = ε·*A*/*t* relation. Polarization *P*(*E*) loops were measured at room temperature in an AixACCT TF Analyzer 3000 platform using triangular *V*(*t*) pulses up to 30 V. The densities of the samples determined from their weight and volume are 5.019 and 5.568 g/cm^3^ for the Nd and Sm compounds, respectively.

### SHG Measurements

Samples were prepared by dispersing onto #1.5-thickness glass coverslips, followed by sealing to ensure mechanical stability. Polarization second-harmonic generation (P-SHG) measurements were performed using a bespoke multiphoton microscope coupled to a femtosecond pulsed laser source (Axon 920, Coherent). Briefly, the excitation beam was directed into an inverted microscope (Eclipse TE2000-U, Nikon) through a beam scanning unit and a half-wave plate for polarization control. The laser beam is focused with an air objective (20× Nikon, NA = 0.75). SHG signals were collected in the forward direction using an air condenser (Nikon, NA = 0.5) and detected with a photomultiplier tube (PMT, Hamamatsu). Spectral separation of the SHG signal from the fundamental excitation laser was achieved using a dichroic mirror (FF670-SDi01, Semrock) in combination with a bandpass filter centered at 460 nm (460/10 nm, Thorlabs) placed before the detector. P-SHG images were acquired by rotating the excitation polarization using the half-wave plate in steps of 10°. Images were recorded over a field of view of 50 μm with a pixel size of 1 μm and an acquisition rate of 1 frame per second. The polarization dependence of the SHG signal was used to infer the crystallographic orientation and confirm the noncentrosymmetric character of the two compounds.

## Results and Discussion

### Synthesis and Structural Study of Nd_2_AlO_3_N and Sm_2_AlO_3_N

The solid state reaction at 1500 °C under N_2_ gas

2 $$\left(1-x\right)R_{2}O_{3}+2x\;\mathrm{RN}+\mathrm{AlN}\to R_{2}\mathrm{Al}O_{3}N\;\left(R=\mathrm{Nd},\mathrm{Sm}\right)$$

produced highly crystalline samples of Nd_2_AlO_3_N and Sm_2_AlO_3_N (see the synchrotron X-ray powder diffraction patterns in Figure 2). The Nd sample was obtained by using a small amount of NdN, *x* = 0.15, which added extra nitrogen to the mixture in order to prevent the formation of the secondary perovskite phase NdAlO_3_, whereas the Sm sample was prepared from Sm_2_O_3_ and AlN (*x* = 0). In both samples, the rare earth nitride RN, the rare earth oxide R_2_O_3_, or the perovskite RAlO_3_ was detected in small proportions in the synchrotron X-ray powder diffraction patterns, as estimated quantitatively from Rietveld refinement. EDX analyses indicated the cationic ratios Nd:Al and Sm:Al of 1.8(4) for Nd_2_AlO_3_N and 1.9(2) for Sm_2_AlO_3_N, and the respective nitrogen contents determined by combustion analysis were 0.92(2) atoms per formula in both cases. Such nitrogen deficiency with respect to the ideal stoichiometry to form the hypothetical solid solution R_2_AlO_3+*x*_N_1–*x*_ would involve (for charge compensation) the existence of reduced oxidation states of the cations, as it has been observed in rare earth tantalum and niobium perovskite-related oxynitrides.^2,10,20^ However, the cations Nd^3+^, Sm^3+^, and Al^3+^ show stable oxidation states and are not expected to be reduced in the present synthesis conditions. The slight deviation from ideal nitrogen content in both samples is understood as being a consequence of secondary oxide phases observed in the synchrotron X-ray diffraction patterns (see Figure 2).

**Figure 2:**
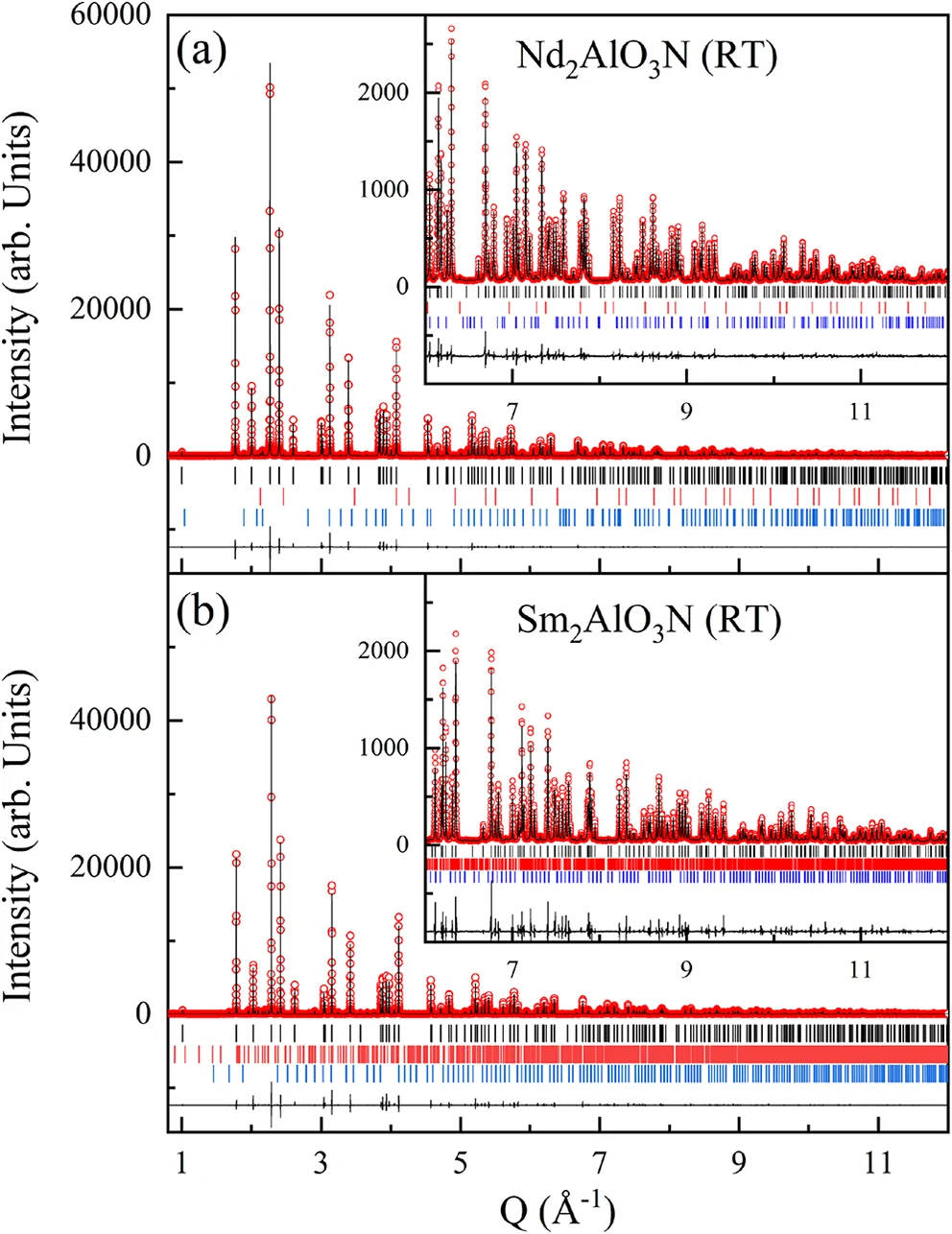
(a) Rietveld fit to room-temperature synchrotron X-ray powder diffraction data (λ = 0.4142 Å) of Nd_2_AlO_3_N performed in the space group *I*4*mm* with cell parameters *a* = 3.703314(3) and *c* = 12.545382(14) Å. Reflection markers correspond to phases Nd_2_AlO_3_N (upper, 97.98% w/w), NdN (0.72% w/w), and Nd_2_O_3_ (1.5% w/w). (b) Rietveld fit to room-temperature synchrotron X-ray powder diffraction data (λ = 0.4138 Å) of Sm_2_AlO_3_N performed in the space group *I4mm* with cell parameters *a* = 3.674477(5) and *c* = 12.40414(3) Å. Reflection markers correspond to the phases Sm_2_AlO_3_N (upper, 92.32% w/w), Sm_2_O_3_ (5.86% w/w), and SmAlO_3_ (1.82% w/w). Insets show the high-Q regions enlarged.

The reconstruction of the reciprocal lattice using electron diffraction led to a tetragonal unit cell with parameters *a* ≈ 3.74 and *c* ≈ 12.60 Å for Nd_2_AlO_3_N and *a* ≈ 3.79 and *c* ≈ 12.65 Å for Sm_2_AlO_3_N (Figure 3), with reflection conditions compatible with the space groups *I*4/*mmm* and *I*4*mm* (*hkl*, *h* + *k* + *l* = 2*n*; *h*00, *h* = 2*n*; 0*k*0, *k* = 2*n*; 00*l*, *l* = 2*n*; *hk*0, *h* + *k* = 2*n*; 0*kl*, *k* + *l* = 2*n*, and *h*0*l*, *h* + *l* = 2*n*). A combined Rietveld refinement of Nd_2_AlO_3_N using synchrotron X-ray and neutron powder diffraction data (Figures 2a and 4, respectively) was performed in *I*4/*mmm* and *I*4*mm* space groups starting with the structural model of the K_2_NiF_4_ archetype for the former and the coordinates reported by Marchand et al.^30^ for the latter. For the noncentrosymmetric space group, the distribution of nitrogen and oxygen in the initial model was considered statistically disordered in the three available anion sites, constraining the anion composition to the nominal stoichiometry. The agreement factors for neutron diffraction data were lower in the *I*4*mm* model (*R*_Bragg_ = 3.03%) compared to the *I4*/*mmm* refinement (*R*_Bragg_ = 3.27%), whereas they were similar for the synchrotron X-ray diffraction data (1.92 and 1.97%, respectively). Independent refinement of anion occupancies for both apical sites of Nd_2_AlO_3_N in the *I4mm* model led to a larger Bragg *R* factor for the neutron diffraction data (3.33%) compared with the refinement constraining the O:N stoichiometry to ideal 3:1. Refinement results for *I4mm* shown in Table 1 and Figure 1c indicate 100% of oxygen population in equatorial sites 4b of the AlX_6_ octahedra with *d*(Al–O1) = 1.8517(1) Å and partial order of both anions in the two crystallographically independent 2b axial sites, where they have relative populations O/N 68/32 and 32/68 with the respective bond distances *d*(Al-X2) = 2.185(15) Å and d­(Al-X3) = 2.034(15) Å. A refinement in *I*4*mm* using anion fixed occupancies with oxygen in 4b sites, 100% N in the X3 site, and 100% O in the X2 site as reported in ref 30, led to a similar agreement *R*_Bragg_ factor for the synchrotron diffraction data (1.89%) but to a larger one for neutron data compared with our model (3.38% vs 3.03%). The differences between the anion distributions observed in this work and the previous neutron diffraction study of Nd_2_AlO_3_N could be a consequence of the different temperature of synthesis of both samples (1500 and 1350 °C, respectively). As indicated above, other perovskites, such as LaTaON_2_, show different N/O ordering in samples prepared by distinct synthetic approaches.^10^

**Figure 3:**
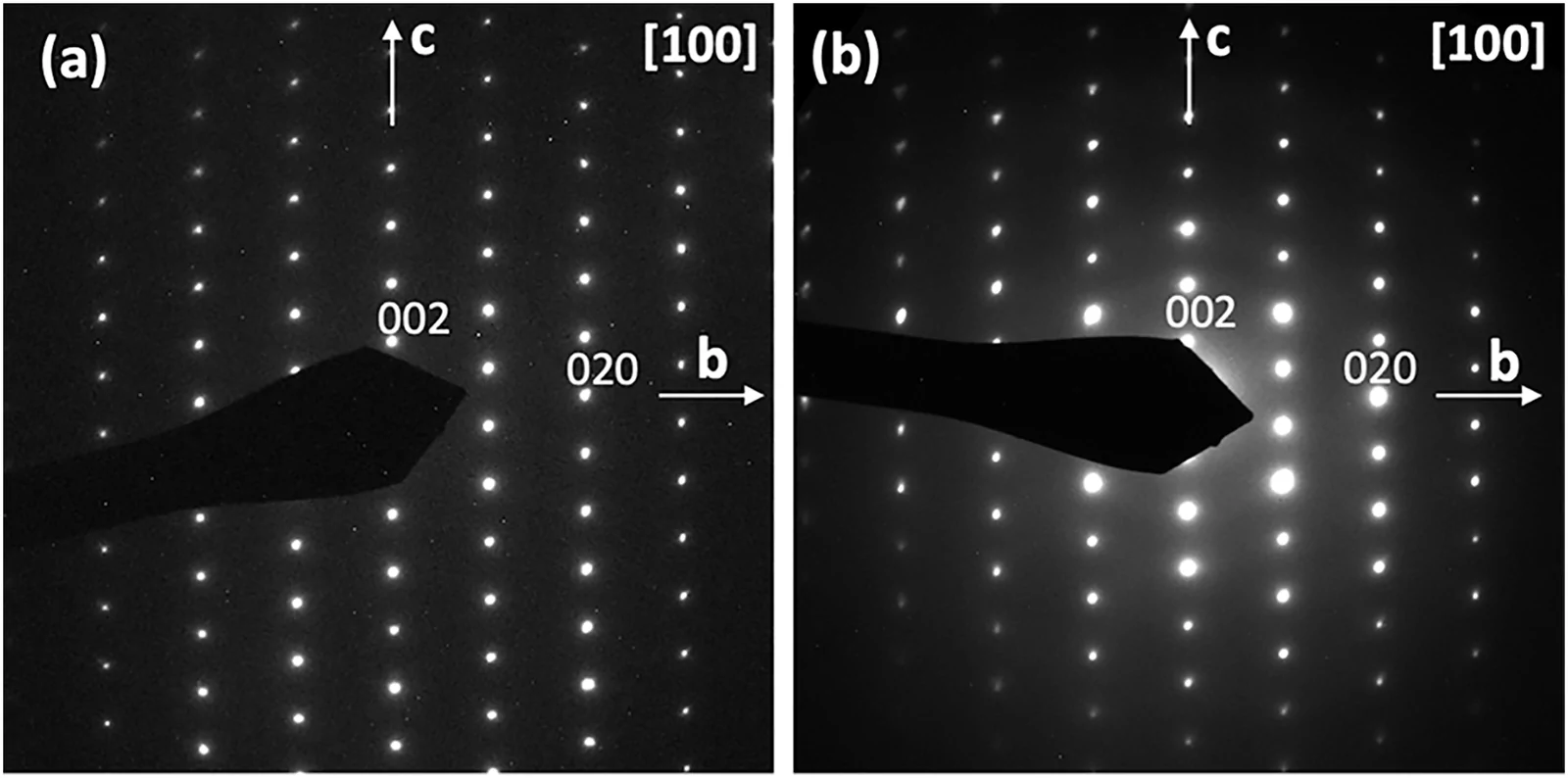
Electron diffraction micrographs of Nd_2_AlO_3_N (a) and Sm_2_AlO_3_N (b) along the zone axis [100].

**Figure 4:**
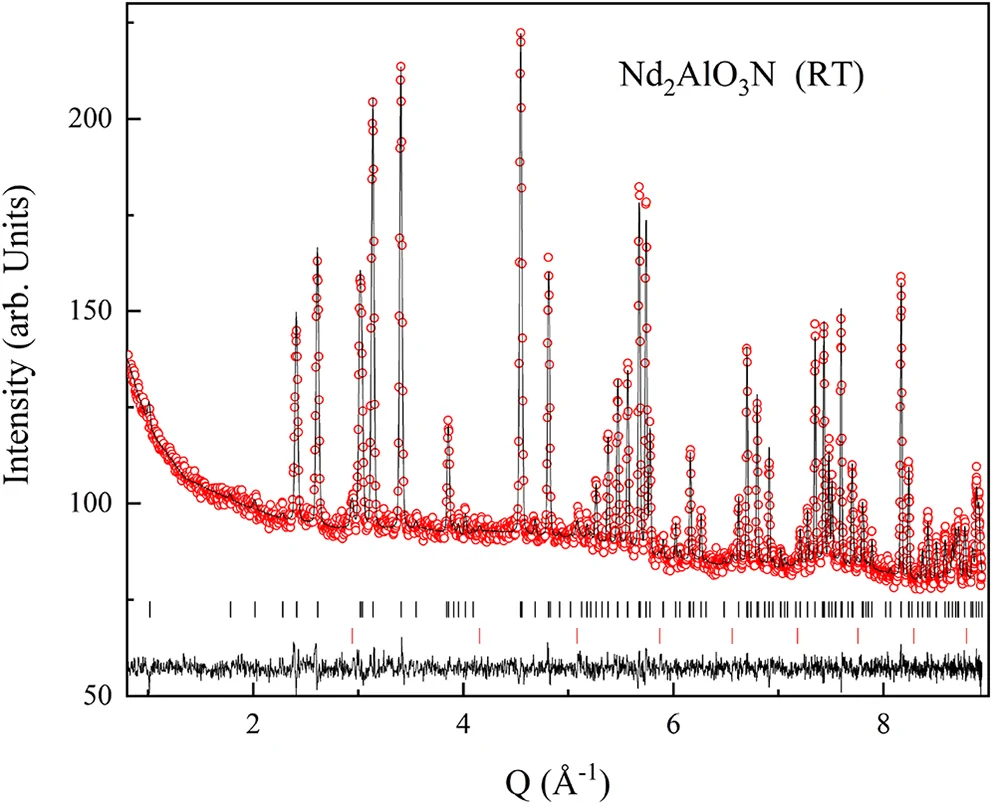
Rietveld fit to room temperature neutron powder diffraction data (λ = 1.358 Å) of Nd_2_AlO_3_N performed in the space group *I*4*mm* with cell parameters *a* = 3.703314(3) and *c* = 12.545382(14) Å. Reflection markers correspond to the phases Nd_2_AlO_3_N (upper) and vanadium from the sample environment.

**Table 1:** Summary of the *I4mm* Model Refined from Room-Temperature Synchrotron X-ray and Neutron Powder Diffraction Data (λ = 0.4147 and 1.358 Å, Respectively) for Nd_2_AlO_3_N^a^,^b^

Atom	Site	*x*	*y*	*z*	B (Å^2^)	Occupancy
Al	2a	0	0	0	0.31(4)	1
Nd1	2a	0	0	0.3502(5)	0.42(5)	1
Nd2	2a	0	0	0.6389(5)	0.40(5)	1
O1	4b	0	0.5	0.9988(1)	0.38(4)	1
O2/N2	2a	0	0	0.8261(12)	0.52(3)	0.68/0.32(13)
O3/N3	2a	0	0	0.1621(12)	0.52	0.32/0.68

Bond	*d* (Å)	Bond	*d* (Å)	Bond	*d* (Å)
Al–O1	1.8517(1) × 4	Al–O2, N2	2.182(15)	Al–O3, N3	2.034(15)
Nd1–O1	2.628(10) × 4	Nd1–O2, N2	2.6360(19) × 4	Nd1–O3, N3	2.360(16)
Nd2–O1	2.553(10) × 4	Nd2–O2, N2	2.348(16)	Nd2–O3, N3	2.6348(18) × 4

a Estimated standard deviations in parentheses are shown once for each independent variable. The z parameter of Al was constrained to 0. Average bond distances: Nd1–O,N = 2.602 Å and Nd2–O,N = 2.567 Å.

b Refined cell parameters are *a* = 3.703314(3), *c* = 12.545382(14) Å, and *V* = 172.0541(3) Å^3^. The corresponding agreement factors for both data are *R*_Bragg_ = 1.92% and *R*_wp_ = 7.36% (synchrotron X-ray powder diffraction data) and *R*_Bragg_ = 3.03% and *R*_wp_ = 1.59% (neutron powder diffraction data).

The [AlO_5_N] octahedra are elongated along the *c* axis, leading to shorter equatorial Al–O1 distances and longer axial Al-X2/X3 distances. Comparing the two axial bond distances, the one for the anion position occupied by a larger proportion of oxygen is larger by about 0.15 Å. This difference can be understood considering the larger covalency of Al–N bonds due to the lower electronegativity of nitrogen compared to oxygen. The elongation of the Al octahedra has also been observed in the related K_2_NiF_4_-type centrosymmetric *I*4/*mmm* oxides SrRAlO_4_ (R = Sm, Nd, La),^34^ where Sr and the rare earth cations are disordered at the A sites, and in the solid solution Sr_1–*x*_La_1+*x*_Al_1–*x*_Mg_*x*_O_4_ (0 ≤ *x* ≤ 0.7).^35^ For SrNdAlO_4_, the observed distances are *d*(Al–O_eq_) = 1.862 Å and *d*(Al–O_ax_) = 2.049(5) Å. This distortion increases the tolerance factor and occurs to stabilize the structure because it favors the adaptability between the perovskite and NaCl-type layers of the *n* = 1 Ruddlesden–Popper structure.^34–36^

The crystal structure of Sm_2_AlO_3_N was refined from synchrotron X-ray diffraction data (Figure 2b) in the space groups *I*4*mm* and *I*4/*mmm*. Neutron diffraction data of this sample were not acquired because the large absorption section of samarium would make it difficult to obtain accurate structural information. The obtained Bragg R factors were similar for both space groups; however, STEM and SHG results (see below) indicated that the structure is noncentrosymmetric. Results of the refinement in the *I*4*mm* space group are shown in Table 2. The anion occupancies were constrained to those of Nd_2_AlO_3_N because X-ray diffraction does not provide enough contrast between the two anions. The observed Al–O,N bond distances were similar to those of the Nd compound, and the Sm–O,N bond distances were slightly lower, as well as the unit cell parameters, as expected from the smaller ionic radius of Sm^3+^.^37^

**Table 2:** Summary of the *I4mm* Model Refined from Room-Temperature Synchrotron X-ray Powder Diffraction Data (λ = 0.4138 Å) for Sm_2_AlO_3_N^a^,^b^

Atom	Site	*x*	*y*	*z*	B(Å^2^)	Occupancy
Al	2a	0	0	0	0.24(4)	1
Sm1	2a	0	0	0.3497(8)	0.46(6)	1
Sm2	2a	0	0	0.6394(8)	0.37(5)	1
O1	4b	0	0.5	0.9896(4)	0.39(8)	1
O2/N2	2a	0	0	0.825(5)	0.40(7)	0.68/0.32
O3/N3	2a	0	0	0.162(5)	0.40	0.32/0.68

Bond	*d* (Å)	Bond	*d* (Å)	Bond	*d* (Å)
Al–O1	1.84 × 4	Al–O2, N2	2.17(7)	Al–O3, N3	2.01(7)
Sm1–O1	2.527(8) × 4	Sm1–O2, N2	2.616(8) × 4	Sm1–O3, N3	2.33(7)
Sm2–O1	2.613(8) × 4	Sm2–O2, N2	2.30(7)	Sm2–O3, N3	2.613(7) × 4

a Estimated standard deviations in parentheses are shown once for each independent variable. The *z* parameter of Al was constrained to 0. Average bond distances: Sm1–O,N = 2.545 Å and Sm2–O,N = 2.578 Å.

b Refined cell parameters are *a* = 3.674477(5), *c* = 12.40414(3) Å, and *V* = 167.4780(4) Å^3^, and agreement factors are *R*_Bragg_ = 2.75% and *R*_wp_ = 13.4%.

The integrated differential phase contrast-scanning transmission electron microscopy (iDPC-STEM) analysis of the samples confirms the noncentrosymmetric character of the Nd_2_AlO_3_N and Sm_2_AlO_3_N structures. iDPC-STEM^38^ enables atomic-resolution imaging of both light- and heavy-element sublattices, allowing quantitative analysis of atomic-column positions and interplanar spacings. Figure 5a shows the iDPC-STEM image of a Nd_2_AlO_3_N crystal viewed along the *a*-axis where Nd, Al, and O/N sublattices are visible. A simple analysis of a perovskite block of the Nd_2_AlO_3_N structure (Figure 5b) and the corresponding intensity profiles measured from the center of the Al atomic column (Figure 5c) evidence the asymmetry in the Al–(O,N) axial bond distances. Figure 5d shows the Al–O/N axial distance map of the iDPC-STEM image, which reveals distinct top and bottom distances, consistent with a polar distortion. However, as shown by the bond length histograms of Figure 5e, the distribution of the top and bottom Al–(O,N) distances exhibits noticeable broadening and partial overlap, indicating a significant local dispersion of bond lengths. The same structural analysis was also performed on a Sm_2_AlO_3_N crystal (Figure 6). In this case, the resulting top and bottom Al–(O,N) axial distance distributions are more clearly separated, suggesting a higher degree of O/N ordering in the Sm compound. The noncentrosymmetric character of both Nd_2_AlO_3_N and Sm_2_AlO_3_N is further supported by the rare-earth axial distance analysis shown in Figure S2.

**Figure 5:**
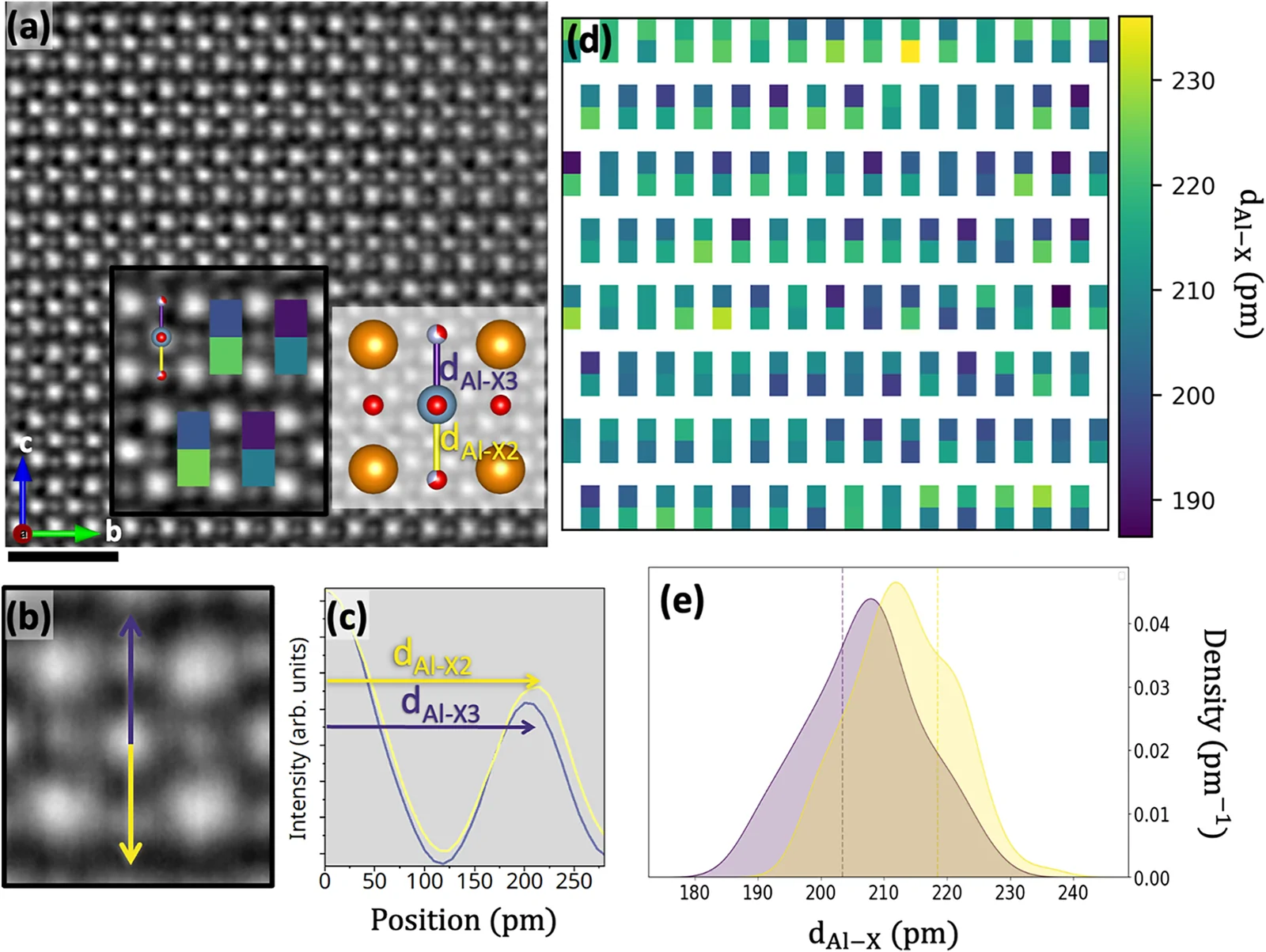
(a) iDPC-STEM image of Nd_2_AlO_3_N viewed along the [100] zone axis, where the Nd, Al, O, and N atomic columns are visible. The inset shows a schematic representation (orange spheres correspond to Nd, blue corresponds to Al, red corresponds to O, and light gray corresponds to N), highlighting the two distinct distances between Al and the apical O/N sites. Scale bar: 1 nm. (d) Distance map of the Al–O/N sites calculated using the center of mass difference between the Al cations and O/N anions from (a). (b) Magnified view of the Nd_2_AlO_3_N structure and (c) corresponding intensity profiles measured from the center of the Al atomic column. (e) Smoothed histograms of the top and bottom axial Al–O/N distances. The vertical bars in (e) correspond to the values measured by neutron diffraction (Table 1).

**Figure 6:**
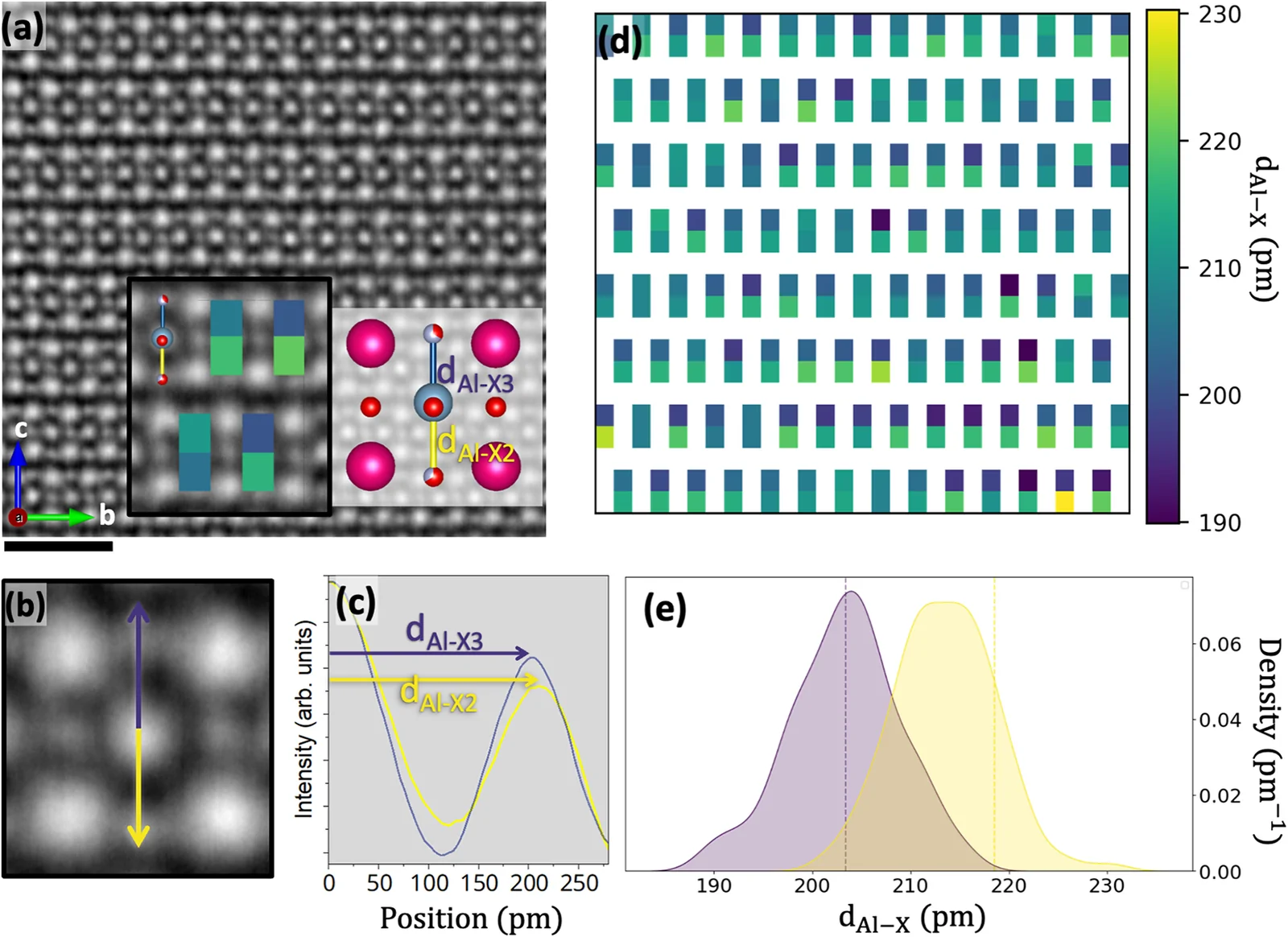
(a) iDPC-STEM image of Sm_2_AlO_3_N viewed along the [100] zone axis, where the Nd, Al, O, and N atomic columns are visible. The inset shows a schematic representation (pink spheres correspond to Sm, blue corresponds to Al, red corresponds to O, and light gray corresponds to N), highlighting the two distinct distances between Al and the apical O/N sites. Scale bar: 1 nm. (d) Distance map of the Al–O/N sites calculated from (a). (b) Magnified view of the Sm_2_AlO_3_N structure and (c) corresponding intensity profiles measured from the center of the Al atomic column. (e) Smoothed histograms of the top and bottom Al–O/N distances, revealing partial overlap together with a systematic shift in their distributions. The vertical bars in (e) correspond to the values measured by X-ray diffraction (Table 2).

The higher degree of O/N ordering in Sm_2_AlO_3_N observed in the STEM images could be rationalized considering the sizes of the two rare earth cations, rather than electronic effects. Indeed, the 4f levels are highly localized and one should not expect important differences in R-(O,N) bonding along the lanthanide series.^39^ However, the decrease of ionic radius of the rare earth should produce an increase of the lattice energy of the oxynitride, a trend that has been clearly stablished for the sesquioxides R_2_O_3_.^40,41^ In Nd_2_AlO_3_N and Sm_2_AlO_3_N, the lattice energy is expected to increase with anion order, which may further stabilize the structure of the Sm compound in a larger degree than in the Nd compound because of the smaller size of Sm^3+^. A different degree of order in Nd_2_AlO_3_N and Sm_2_AlO_3_N samples could also be caused by the distinct synthesis conditions used for the two compounds. As indicated above, our sample of Nd_2_AlO_3_N shows a lower degree of O/N ordering than the sample prepared by Marchand et al. at lower temperature.^30^ Our samples of Nd_2_AlO_3_N and Sm_2_AlO_3_N were both prepared at 1500 °C, but the Nd sample was treated at this temperature for a longer time than the Sm one (48 h versus 12 h), which could induce a higher degree of O/N disorder in the former.

### Optical Properties

In Figure 7a,b, we display the wavelength-dependent reflectivity spectra of Nd_2_AlO_3_N and Sm_2_AlO_3_N, respectively. Well-defined absorption edges can be identified in both samples, and the characteristic absorption bands associated with 4f–4f transitions can be observed above the absorption edge. The absorption bands occur at energies that nicely coincide with the typical energies reported for Nd^3+^ and Sm^3+^ rare-earth oxides and correspond to the transitions indicated.^42^ For each oxynitride, the relative intensities of the different absorption bands are in agreement with the corresponding oscillator strengths (*f*_osc_) reported in similar materials. It is worth noticing that the absorption bands in the Sm compound are well-defined. This is a striking observation as the predicted *f*_osc_ strength for Sm^3+^ is much smaller than that predicted for Nd^3+^ cations, both in the same crystalline environment.^42,43^ Figure 7c,d depicts the Tauc plot (*F*(*R*)­ℏν^2^) around the well-defined absorption edge region in both samples, where the frequency-dependent diffuse reflectance (*R*(ν)) is used to calculate the Kubelka–Munk function (defined as *F*(*R*) = (1 – *R*)^2^/2*R*) and ν is the frequency of light. It is observed that the absorption edge is unambiguous. Using the Tauc plots, graphed here for direct transitions, we extract an optical band gap of 2.25 eV for Nd_2_AlO_3_N and 2.45 eV for Sm_2_AlO_3_N in agreement with the orange and yellow-green colors that are shown by the samples. These values are in reasonable agreement with results from Cheviré et al.,^29^ who observed a valence band to conduction band type absorption at about 525 nm (2.36 eV) and 485 nm (2.55 eV) in the diffuse reflection spectra of the Nd and Sm compounds, respectively.

**Figure 7:**
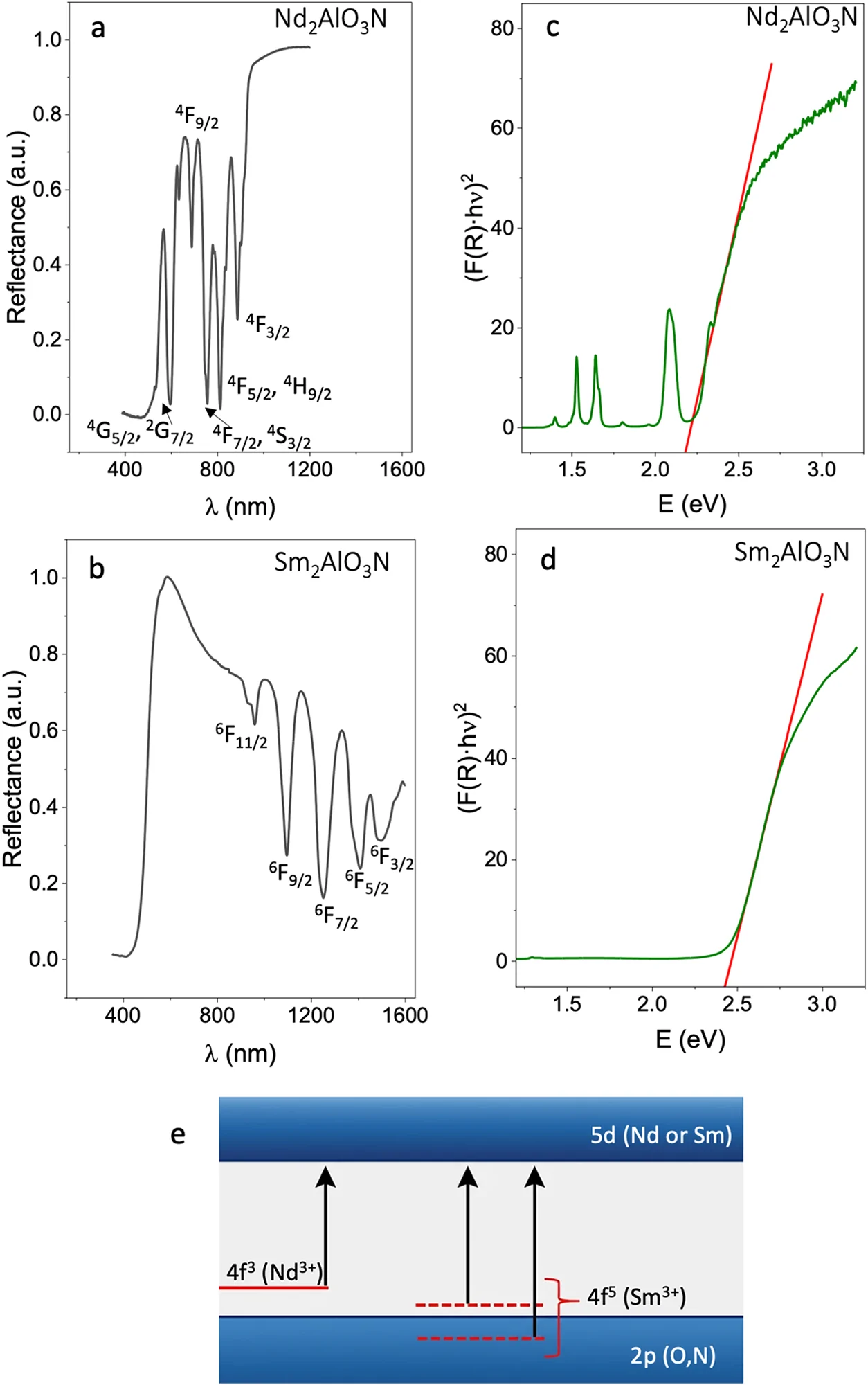
Diffuse reflection spectra and Kubelka–Munk plots for a direct band gap transition of Nd_2_AlO_3_N (a, c) and Sm_2_AlO_3_N (b, d). In (a, b), the energy level assignments for the transitions from ^4^I_9/2_ and ^6^H_5/2_ states of Nd^3+^ and Sm^3+^, respectively, are indicated. (e) Schematic energy diagram depicting the 4f levels and 5d and 2p bands for Nd_2_AlO_3_N and Sm_2_AlO_3_N.

The optical band gap dependence on the rare earth-containing crystalline oxides has been extensively explored, both experimentally^44,45^ and theoretically.^46–50^ In the sesquioxides Sm_2_O_3_ and Nd_2_O_3_, the optical band gap is somewhat larger for Sm than for the Nd compound (for instance, 5 and 4.7 eV, respectively) (Gillen et al.^47^ and references therein). The same trend is observed in the oxynitrides Sm_2_AlO_3_N and Nd_2_AlO_3_N, although both band gaps are smaller. The conduction band in the sesquioxides is primarily formed by 2p-O orbitals, while the bottom of the conduction band is basically formed by empty 5d orbitals of the rare earth. In the oxynitrides, the smaller electronegativity of N compared to O pushes up and broadens the 2p­(N,O) bands, thus producing a narrowing of the optical band gap in comparison to the oxides. The observation of a somewhat smaller band gap in the Sm oxynitride compared with the Nd compound indicates that the optical gap is affected by the 4f*^n^* configuration of the rare earth. Sm^3+^ is a 4f^3^ ion and Nd^3+^ is a 4f^5^ ion, and thus, the ionization energy of the latter is larger than the former as the corresponding Z number is larger. This gives rise to the common trend of lowering (deepening) the energy of 4f*^n^* orbitals as n increases. As a consequence, the experimental data in the two oxynitrides indicate that the position of the 4f*^n^* energy levels of the rare earth ultimately dictates their optical response. The experimental data can be rationalized by assuming that the band gap in Nd_2_AlO_3_N is dictated by the 4f^3^ → 5d-based conduction band, as illustrated in Figure 7e, whereas in Sm_2_AlO_3_N, photon absorption is governed by either 4f^5^ → 5d or 2p­(O,N) → 5d, depending on if the 4f^5^ remains in the band gap or has been pushed down below the VB edge by its stronger nuclear attraction (dashed lines in Figure 7e). In fact, a similar scenario has been proposed for the sesquioxides R_2_O_3_.^47^ More precisely, in the sesquioxides, the difference in binding energy between 4f^3^ and 4f^5^ is about 1 eV.^46,51^ A closely similar energy difference is expected to hold in the R_2_AlO_3_N compounds. If so, as the observed band gap difference between Nd_2_AlO_3_N and Sm_2_AlO_3_N is only 0.3 V, it follows that the 4f^5^ state should lie below the 2p-(O,N) valence band edge, and thus, the band gap is mainly dictated by 2p­(O,N) → 5d transitions. Therefore, in Sm_2_AlO_3_N, and in contrast to Sm_2_O_3_,^47^ the wider 2p­(O,N) valence band absorbs the 4f^5^ states below this edge and changes the nature of optical absorption compared to Nd_2_AlO_3_N.

### Second-Harmonic Generation Behavior

To assess the noncentrosymmetric character of the samples, polarization second-harmonic generation spectroscopy imaging was performed. Figure 8 shows that for both compounds, only a subset of crystals exhibits a detectable SHG signal, which is attributed to the orientation dependence of the nonlinear optical response. Specifically, effective SHG generation occurs when the crystalline axes are favorably aligned with respect to the polarization of the excitation beam. Additionally, the particles display SHG polarization dependence, which indicates differences in their crystallographic orientations. These observations are consistent with the random distribution and orientation of the particles upon their deposition. The presence of a polarization-dependent SHG signal confirms the noncentrosymmetric structure of both Nd_2_AlO_3_N and Sm_2_AlO_3_N.

**Figure 8:**
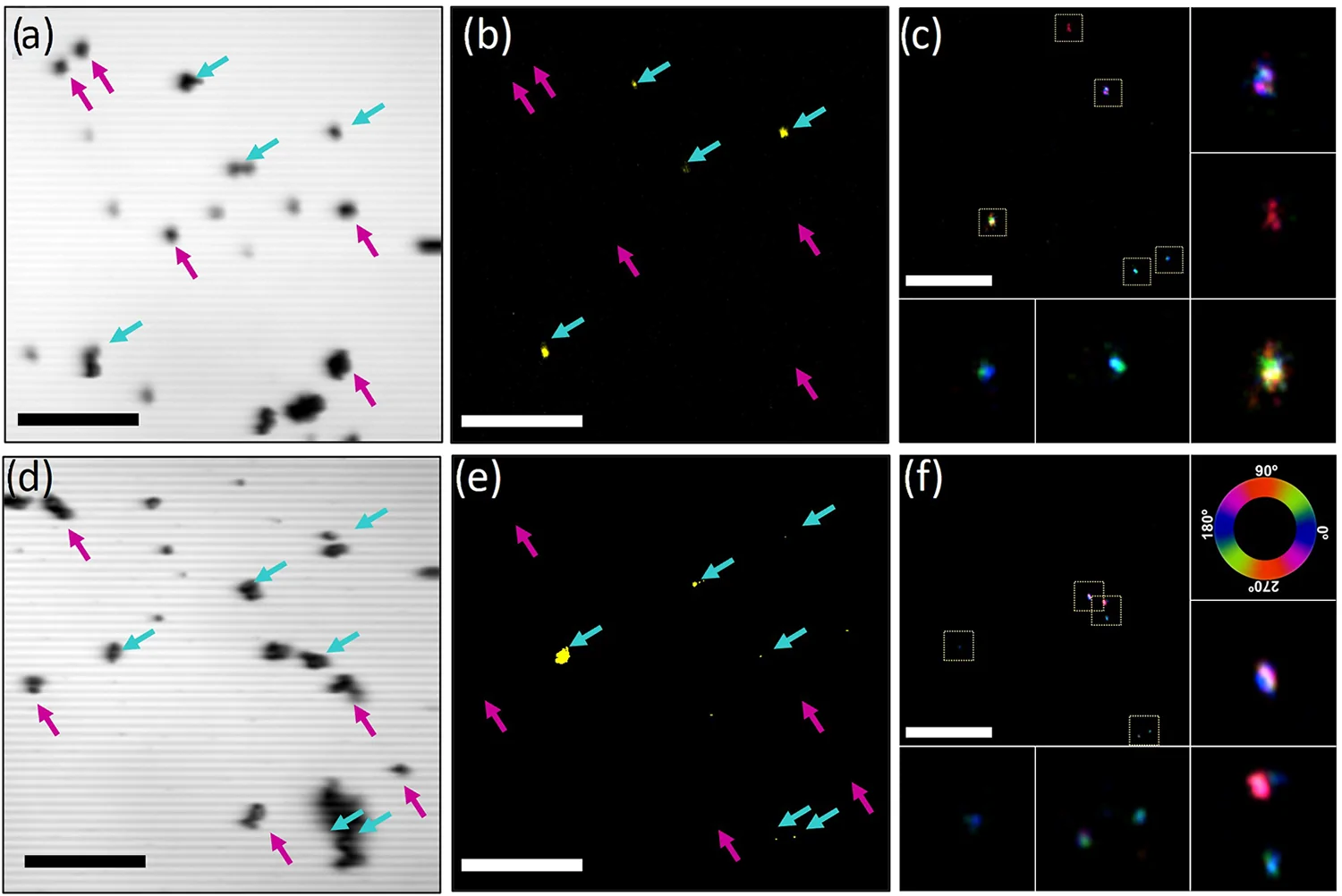
P-SHG imaging of the samples. (a), (d) Transmission images of dispersed particles of Sm_2_AlO_3_N and Nd_2_AlO_3_N on a glass coverslip, respectively. (b), (e) SHG images of Sm_2_AlO_3_N and Nd_2_AlO_3_N samples, respectively; cyan arrows indicate the particles that produced the SHG. Particles indicated by the pink arrows do not produce SHG because the crystalline axis is not favorably aligned with respect to the polarization excitation beam. (c), (f) Polarization-resolved SHG (P-SHG) images of Sm_2_AlO_3_N and Nd_2_AlO_3_N samples, respectively, where the color encodes the orientation of the excitation polarization corresponding to maximum SHG intensity. Scale bar: 30 μm.

### Dielectric Properties

Frequency-dependent permittivity, measured at 1 V AC signal, (Figure 9a) for Nd_2_AlO_3_N (circular capacitor, 4.77 mm diameter and 810 μm thickness) and Sm_2_AlO_3_N (circular capacitor, 4.90 mm diameter and 860 μm thickness) display a broad frequency range extending from 5 kHz to 5 MHz, where ε_exp_ is rather independent of frequency, indicating an intrinsic dielectric response. In this frequency range, the observed capacitance leads to a relative permittivity (ε_m_) of 180 and 110, respectively. The experimentally determined densities (ρ_exp_) of the used pellets are 5.019 g/cm^3^ (Nd_2_AlO_3_N) and 5.568 g/cm^3^ (Sm_2_AlO_3_N), significantly lower than the corresponding densities deduced from Rietveld-refined structural parameters (ρ_ref_ = 7.286 and 7.730 g/cm^3^), which indicate a notable porosity of the samples. Therefore, the intrinsic permittivity (ε_r,int_) is estimated by correcting the measured ε_r_ values by the density ratio: ε_r, int_ = ε_r_·(ρ_ref_/ρ_exp_), which leads to 260 and 150 for Nd_2_AlO_3_N and Sm_2_AlO_3_N, respectively. For Nd_2_AlO_3_N, permittivity data in Figure 9 show a small decrease with frequency, which arises from the contribution of a leakage-related resistive channel in the RC (resistance capacitance) equivalent circuit of the measured sample.^52^ For Sm_2_AlO_3_N, dependence on frequency is less pronounced, indicating a reduction of the leakage-related current contribution. In both samples, at high frequency, ε_r_ diverges as a consequence of the series resistance contribution arising from the contacts and/or wiring resistances.^53^

**Figure 9:**
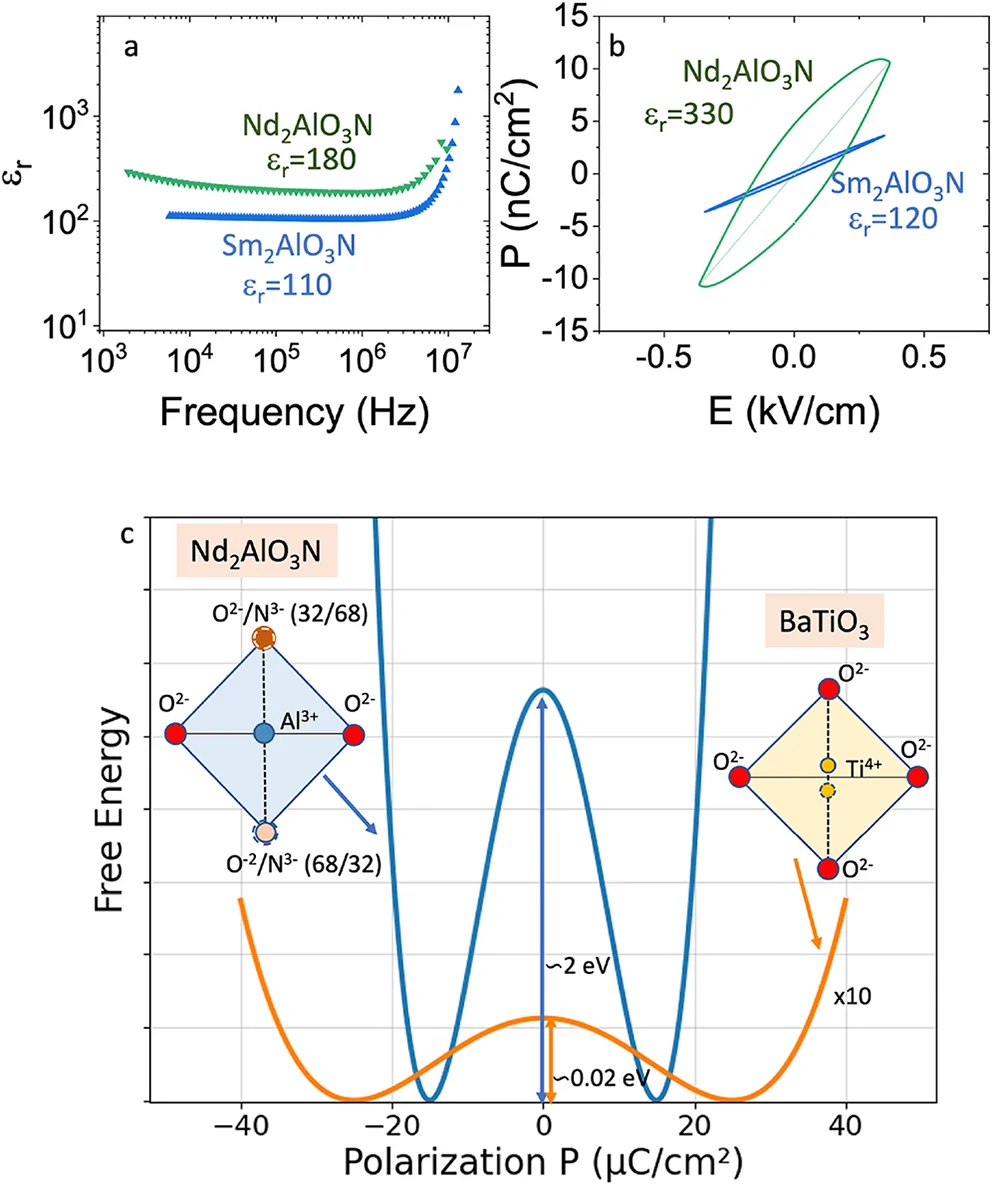
(a) Frequency-dependent ε_r_ for Nd_2_AlO_3_N and Sm_2_AlO_3_N samples. (b) *P*–*E* loops for Nd_2_AlO_3_N and Sm_2_AlO_3_N samples. (c) Sketch of the free energy of Nd_2_AlO_3_N and a prototypical soft ferroelectric (e.g., BaTiO_3_) is included for comparison. The energy barriers mainly dictating the coercive field are indicated.

Figure 9b shows P–E loops collected at 1 kHz. We first note that while both loops display some hysteresis, the loop recorded for Sm_2_AlO_3_N is almost reversible, while this is not the case for Nd_2_AlO_3_N, where a pronounced hysteresis is observed, which masks its genuine dielectric response. This is due to leakage currents in the sample, more pronounced in the latter. The average slope of the P­(E) loop (120 for Sm_2_AlO_3_N, 320 for Nd_2_AlO_3_N) is a measure of the sample relative permittivity (in the measured field range). These values are somewhat larger than those obtained from the permittivity measurements shown in Figure 9a. This is due to the fact that in this case (Figure 9b), the applied ac signal is up to 30 V, much larger than the 1 V used for AC permittivity data in Figure 9a, and leakage currents are known to be exponentially increasing with applied fields. Dielectric losses evaluated by tan δ were below 0.05, as limited by our experimental resolution, for both samples across the entire frequency range explored. The observation of a large permittivity is found for other perovskite oxynitrides: for instance, ε_r_ ≈ (200–220) has been observed in BaWON_2_ and has been ascribed to electrical dipoles induced by anion order and the second-order Jahn–Teller effect in W^6+^ cations.^16^ Here, the high ε_r_ values can be similarly attributed to the presence of (O/N)-Al-(O/N) electrical dipoles lying along the c direction, with an enhanced polarizability due to the inherent (O/N) disorder at each apical octahedral position. As stated before, from the observation of STEM images, such disorder is more important in Nd_2_AlO_3_N compared to Sm_2_AlO_3_N, which may contribute to an additional enhancement of ε_r_ in the former, in agreement with the reported influence of local polar disorder and structural inhomogeneities on the dielectric response of disordered polar materials.^54,55^ A more interesting question is if polar Nd_2_AlO_3_N and Sm_2_AlO_3_N oxynitrides are ferroelectric or not, as in the P­(E) loops of Figure 9b, ferroelectric switching is not observed. Note that to observe switching, the polarization should overcome an energy barrier under the presence of a large enough electric field (the coercive field). In an archetypical ferroelectric such a BaTiO_3_, this energy barrier is relatively small (≈20 meV) (Figure 9c) due to the minute shift of the small 3d^0^-Ti^4+^ ions out of the center of symmetry at the displacive ferroelectric–paraelectric transition.^56^ Correspondingly, the coercive field is relatively small (10^3^–10^4^ V/cm). In Figure 9b, the largest applied field (0.5 kV/cm) may be smaller than the coercive field. However, inspection of the structure indicates that polarization reversal, for instance, O^2–^/N^3–^ (32/68)-Al^3+^-O^2–^/N^3–^ (68/32) to its reverse state, would require anion migration, so it is expected to be diffusion-limited. Energies for diffusive ionic motion, common in defective solid oxides, are typically larger than tenths of eV, largely depending on the structure and the diffusion path. For Ruddlesden–Popper oxides, the reported energies are within the 1–2 eV range.^57^ This energy would imply a large energy barrier for polarization switching and thus a prohibitive large coercive field, as illustrated in Figure 9c. It follows that most likely, the polar nature of these anion-ordered oxynitrides is not switchable by an electric field. The situation is similar to that occurring on Na_3_MoO_3_F_3_, where the polar units [MoO_3_F_3_]^−3^ are not switchable unless breaking the structure. Recently, Poeppelmeier, Rondinelli, and co-workers have reported first principle studies of possible mechanisms of polarization reversal in KNaNbOF_5_,^58^ where switching may occur due to uncompensated antipolar displacements of oxygen and fluorine and does not require oxygen and fluorine site exchange; more recently, they have theoretically explored a possible mechanism for switching in anion-ordered Ruddlesden–Popper oxyfluorides (e.g., Sr_2_ScO_3_F and Ca_2_ScO_3_F).^59^ The authors concluded that in this case, anion substitution leads to some buckling of the ScO_5_F octahedra that brings in polarization: “improper anion order ferroelectricity”. The energy barrier for buckling being relatively small would allow polarization switching. Unfortunately, the absence of octahedral buckling in the present oxynitrides does not allow a low-energy barrier for switching. We thus conclude that Nd_2_AlO_3_N and Sm_2_AlO_3_N should be polar pyroelectrics.

## Conclusions

The compounds Nd_2_AlO_3_N and Sm_2_AlO_3_N have been prepared using a new synthesis pathway by solid-state reaction under flowing N_2_ at 1500 °C of mixtures of oxides and nitrides. They crystallize in the *I*4*mm* space group representing the only examples of polar Ruddlesden–Popper oxynitrides and one of the few cases of noncentrosymmetric oxynitride perovskite-related compounds. Powder neutron diffraction data of Nd_2_AlO_3_N indicate partial N/O order in the two axial positions X2 and X3 of AlO_5_N octahedra, with average nitrogen occupancies of 32% and 68%, respectively, and Al-X distances of 2.182(15) and 2.034(15) Å, respectively. The anion disorder in these positions is predicted from bond strength calculations using the Pauling second crystal rule. The difference between the two axial bond distances can be directly observed in the STEM iDPC images of both Nd and Sm compounds, resulting in local dipoles that show some disorder along the crystal, in agreement with the observed N/O distribution from neutron diffraction data of Nd_2_AlO_3_N. The polar nature of both compounds is accompanied by large dielectric permittivity (≈260 and 150 for Nd_2_AlO_3_N and Sm_2_AlO_3_N, respectively). The somewhat larger permittivity of Nd_2_AlO_3_N could be assigned to the enhanced local disorder of dipoles, as revealed by STEM iDPC imaging. The optical band gaps are found to be 2.36 and 2.55 eV for the Nd and Sm compounds, respectively, which are smaller than in the corresponding rare-earth sesquioxide due to the higher valence-band edge of 2p­(O/N) compared to 2p­(O). The relative narrower band gap in Nd_2_AlO_3_N compared to the Sm_2_AlO_3_N compound is attributed to the nature of optical transition in both compounds where the fine details are governed by the position of the corresponding 4f states of Nd^3+^ and Sm^3+^ ions relative to the 2p­(O/N) valence band edge. SHG has provided a conclusive demonstration of the polar nature of both compounds, which we argue are not switchable, and thus, they are not ferroelectric but pyroelectric. These results increase the diversity of properties in the group of perovskite-related oxynitrides and open new possibilities to explore polar heteroanionic materials.

## Supplementary Material



## Notes

Workflow of the calculation of interatomic distances from iDPC images of Sm_2_AlO_3_N and maps of distances along *c* for Nd-X and Sm-X (X = O,N) extracted from iDPC images (PDF)
