# Supplementary material for: Polar Ruddlesden–Popper Rare-Earth Aluminum Oxynitrides with Partial Anion Order
Source: Inorg Chem. 2026 Oct 13;65(42):24918–29. doi: 10.1021/acs.inorgchem.6c03989 (PMC13715573; doi:10.1021/acs.inorgchem.6c03989)
Supplement: Supplementary file 1 [file ic6c03989_si_001.pdf]

Supplementary Information

Polar Ruddlesden-Popper rare earth aluminum oxynitrides with partial anion order

Judith Oró-Solé,[§,#] Jhonatan R. Guarín,[§,#] Ignasi Fina,[§,] Carlos Frontera,[§] Jaume Gàzquez,[§] Saul Estandia,[§] Mehrdad Ghiasabadi Farahani,[§] Clemens Ritter,[‡] Pablo Loza-Alvarez,[†] Gustavo Castro-Olvera,[†] Josep Fontcuberta[§,*] and Amparo Fuertes[§,*]*

§ Institut de Ciència de Materials de Barcelona (ICMAB-CSIC), Carrer dels Til.lers, 08193 Cerdanyola del Vallès, Spain.

‡ Institut Laue-Langevin, 71 Av. de Martyrs, BP 156, F-38042, Grenoble Cedex 9, France.

† ICFO-Institut de Ciències Fotòniques, The Barcelona Institute of Science and Technology, 08860 Castelldefels, Spain.

*Amparo Fuertes: amparo.fuertes@icmab.es

*Josep Fontcuberta: fontcuberta@icmab.cat

*Ignasi Fina: ifina@icmab.es

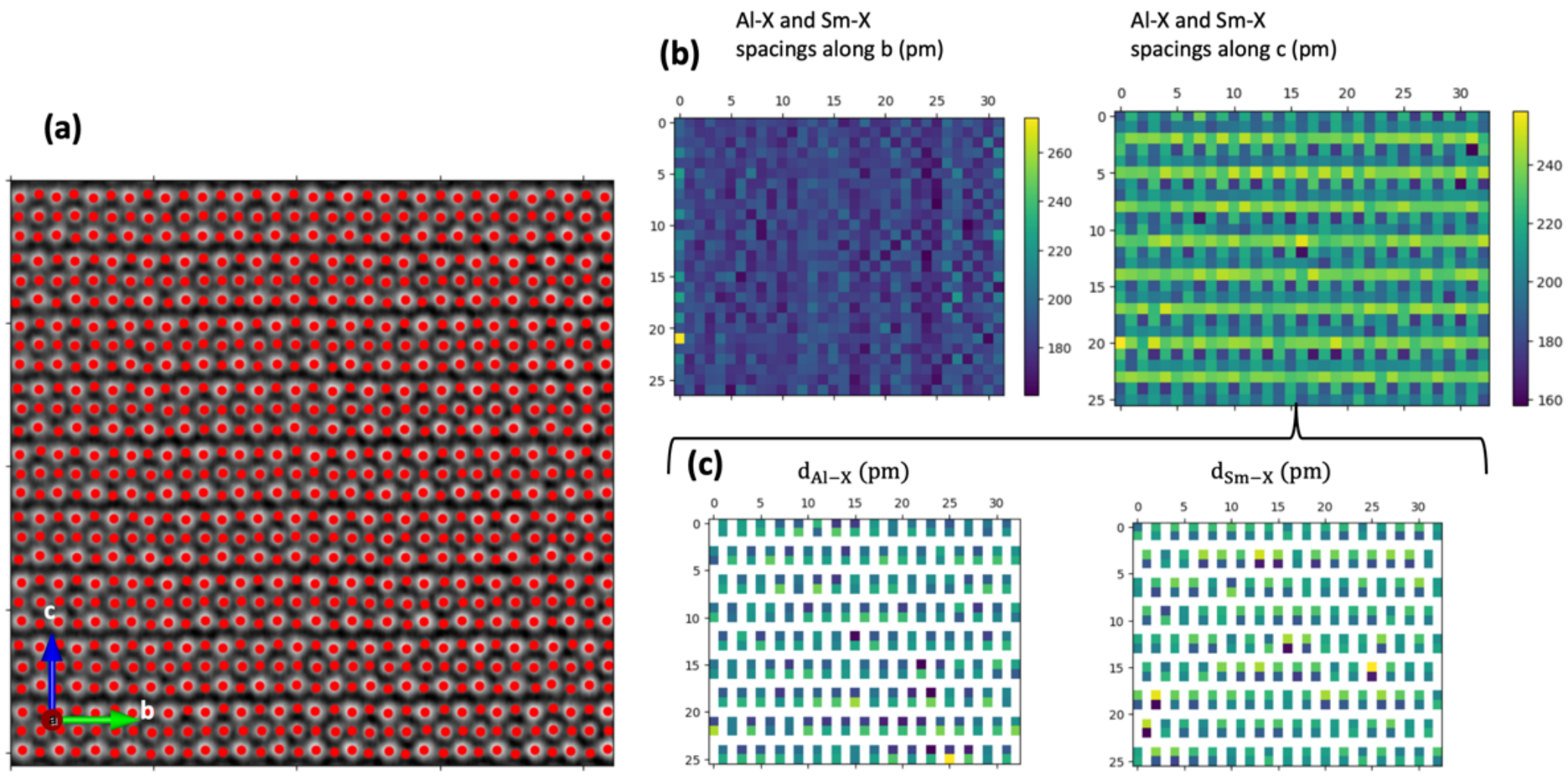

Figure S1. Workflow of the calculation of interatomic distances from iDPC images of Sm_2AlO_3N. (a) iDPC image with superimposed red dots marking the Sm, Al, and O/N atomic column positions. (b) Maps of extracted distances along b and c derived from the position matrix in (a). (c) Masked maps of distances along c separating the distances into Sm–X and Al–X (X= O,N).

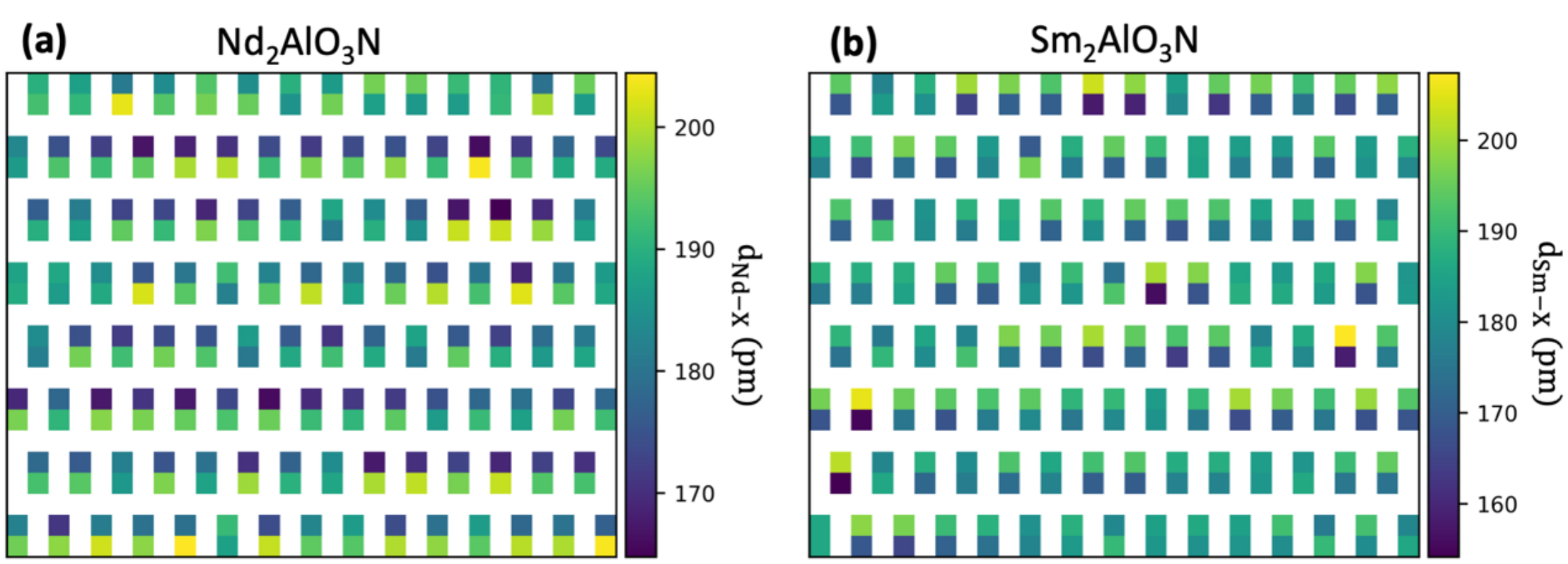

Figure S2. Maps of distances along c for Nd-X (a) and Sm-X (b) (X= O,N), revealing a non-centrosymmetric structure.
